# Detailed Analysis of 17β-Estradiol-Aptamer Interactions: A Molecular Dynamics Simulation Study

**DOI:** 10.3390/molecules23071690

**Published:** 2018-07-11

**Authors:** Alexander Eisold, Dirk Labudde

**Affiliations:** 1 Faculty of Applied Computer and Biosciences, University of Applied Sciences Mittweida, Technikumplatz 17, 09648 Mittweida, Germany; dirk.labudde@hs-mittweida.de; 2 Institute for Organic Chemistry, Technische Universität Bergakademie Freiberg, Leipziger Straße 29, 09599 Freiberg, Germany

**Keywords:** molecular dynamics simulation, 17β-estradiol, DNA-aptamer, GROMACS, modeling

## Abstract

Micro-pollutants such as 17β-Estradiol (E2) have been detected in different water resources and their negative effects on the environment and organisms have been observed. Aptamers are established as a possible detection tool, but the underlying ligand binding is largely unexplored. In this study, a previously described 35-mer E2-specific aptamer was used to analyse the binding characteristics between E2 and the aptamer with a MD simulation in an aqueous medium. Because there is no 3D structure information available for this aptamer, it was modeled using coarse-grained modeling method. The E2 ligand was positioned inside a potential binding area of the predicted aptamer structure, the complex was used for an 25 ns MD simulation, and the interactions were examined for each time step. We identified E2-specific bases within the interior loop of the aptamer and also demonstrated the influence of frequently underestimated water-mediated hydrogen bonds. The study contributes to the understanding of the behavior of ligands binding with aptamer structure in an aqueous solution. The developed workflow allows generating and examining further appealing ligand-aptamer complexes.

## 1. Introduction

**Figure 1:**
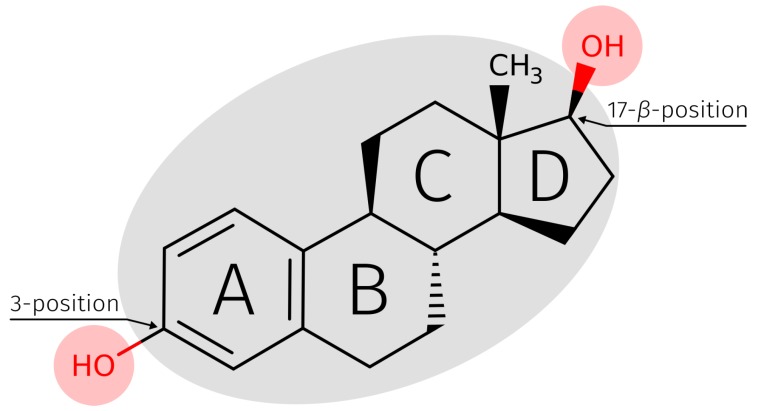
Skeletal formula of E2 with three highlighted structural features. The two red shaded circles are hydroxyl groups at 3- and 17-β-position and can act as donor or acceptor in H-bonds. The grey shaded area represents the hydrophobic rigid backbone. The A-ring of E2 is the aromatic ring system for the formation of possible π-stacking interactions. Skeletal formula of E2 adapted from DrugBank [39]. E2, 17β-estradiol.

Pharmacological active chemicals have been detected in drinking water and other water resources [1,2] and their negative effects on various organisms have been observed [3,4]. 17β-Estradiol (E2), for example, has been detected in low concentrations between 0.1 and 3.6 ng·L${}^{‒1}$ in different water samples [5]. E2 (Figure 1) is the most potent endogenous steroid estrogenic sex hormone in mammals [5]. Exogenous E2 is an endocrine disruptor and has damaging effects on the reproductive health of male humans [6,7]. This leads to concerns, since E2 is present in ground and drinking water [8,9], which makes it necessary to monitor its concentration [6]. Additionally, E2 has been detected in waste water of municipal sewage treatment plants, hospitals, and municipal landfills [10,11]. It was shown that elevated environmental estrogen concentrations have profound effects on fish reproduction, which interrupts the aquatic food chain [12,13]. Furthermore, bioaccumulation of environmental E2 has been observed in several plant species [10]. For the detection or filtration of E2, it is possible to use aptamers, as they have already used for the detection of a large number of small compounds [14].

Aptamers are short single-stranded RNA or DNA oligonucleotides that fold in a complex three-dimensional (3D) structure and bind specific molecules [15]. They specifically recognize small molecules [16,17], proteins [18,19], or even whole bacteria [20,21]. To reliably bind a target molecule, the aptamer needs to adopt a stable 3D structure. Only with a stable structure the ligand can be bound efficiently by means of specific interactions [22]. Ligand-specific aptamers can be extracted by the established in vitro systematic evolution of ligands by exponential enrichment process (SELEX) [15,23]. Besides the artificial aptamers, which originate from randomized libraries and can be determined by SELEX, there are also natural aptamers, e.g., riboswitches. Riboswitches are responsible for many natural binding mechanisms as well as the regulation of gene expression occurring in organisms [24,25]. The SELEX method has become the standard method and was further developed in various directions. For example, one development was the optimization and generation of a targeted start library [26,27]. The specificity of aptamers makes them suitable for the detection of various targets with low concentrations in different environments and, therefore, aptamers can be used as biosensors of substances such as E2 [5,6,16,28]. Svobodová and colleagues compared and validated different E2-specific aptamers. For the validation process, microscale thermophoresis, apta-PCR affinity assay and surface plasmon resonance were used [29]. In this study, was chose the E2-specific 35-mer aptamer identified by Alsager et al. [16]. Several studies have successfully shown that this aptamer binds E2 [16,29]. There are no crystal or NMR structures available for these aptamers with which an analysis of the interaction between E2 and its specific aptamer would be possible. In general, crystal structures of aptamer–ligand complexes are sparse. However they are crucial to understand the basic binding between aptamer and ligand. Nonetheless, it is possible to analyze these binding mechanisms at the molecular level.

Molecular dynamics (MD) simulation is a powerful methodology for the analysis of interactions between a ligand and an target. It is the calculation of movements and interactions of atoms and molecules within a defined time interval in silico [30]. MD simulations have been used for a variety of applications, for example in the computational based investigation of conformational transitions in common microbial enzymes in the presence and absence of single-walled carbon nanotubes [31,32]. The prerequisites for this are existing 3D structure information. The aptamer structure can be defined using modeling software such as the MacroMoleculeBuilder (MMB) [33]. Additionally, a useful alternative to study this binding is the MD simulation method, which has often been used [34,35,36,37]. Hilder and Hodgkiss analyzed binding bases for an E2-specific aptamer and other aptamers [35]. The binding mechanism was examined with the 22-mer E2-specific aptamer [16] and other designed aptamers with the help of MD simulations [35]. However, detailed analyses of the interactions between the E2 and a specific aptamer have not been performed.

In this study, the Protein-Ligand Interaction Profiler (PLIP) tool [38] was used, which made it possible to determine the interactions between E2 and aptamer structure in each time step of the MD simulation. PLIP was originally developed for the detection of interactions between ligands and protein structures, but has been extended to enable the detection of interactions between a ligand and RNA/DNA structure. This allows to analyzed the following interaction types between E2 and the aptamer for each time step: hydrogen bonds (H-bonds), water-mediated H-bonds, π-stacking, and hydrophobic interactions. The underlying binding process between E2 and its specific aptamer has so far only been analyzed in one study [35]. Crystal structures of aptamer–ligand complexes are rare, but crucial for understanding the basic binding between an aptamer and a ligand. By observing the interactions that remained stable over the course of the simulation, it was possible to determine the essential binding atoms. The entirety of those atoms represent the binding site.

## 2. Results

This study presents the analysis of ligand-aptamer interactions between E2 and its specific aptamer. The results show the characterization of the underlying E2-specific aptamer and the investigation of this structure after modeling. Furthermore, two 25 ns MD simulations, aptamer without and with the ligand E2, are examined in detail and compared to each other. Non-covalent interaction analysis between E2 and the aptamer has been performed at the atomic level and also for individual bases of the aptamer. The results obtained contribute to a better understanding of ligand-aptamer binding, in particular by combining the methods presented here.

### 2.1. Generation of the ssDNA Aptamer Structure

**Figure 2:**
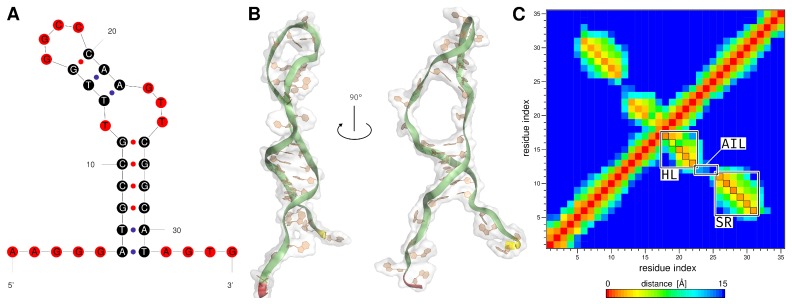
(**A**) 2D structure of the E2-specific aptamer identified by Alsager et al. [16]. Bases highlighted black form base pairs and bases highlighted in red are unpaired. (**B**) Modeled structure of the E2-specific aptamer obtained from MMB [33]. The 5${}^{\prime }$-end is shown in red within the structure and the 3${}^{\prime }$-end in yellow. (**C**) Base distance map for determining the distances between the individual base pairs. Bases that are very close to each other are displayed in red. The SSEs are framed in black. Within the framed areas, the orange fields show the formed SSEs with a distance range between 1.54 Å and 1.92 Å. The basis in the AIL region have a distance between 13.10 Å and 15.00 Å. AIL, asymmetric interior loop; HL, hairpin loop; MMB, MacroMoleculeBuilder; SR, stem region; SSEs, secondary structure elements.

Since no experimentally determined 3D structure of the aptamer exists, it has to be modeled first. Based on the 2D representation of single stranded DNA (ssDNA) (Figure 2A), the 3D structure was generated with MMB [33] (Figure 2B and the corresponding animation is provided in Video S1), which was the starting point of the performed MD simulation. The E2-specific aptamer consists of five unbound bases AAGGG at the 5${}^{\prime }$-end, four unbound bases AGTG at the 3${}^{\prime }$-end, and the following secondary structure elements (SSEs): a stem region with six intramolecular base pairs, an asymmetric interior loop and a stem-loop (hairpin loop) (Figure 2A). A distance map (Figure 2C) can be used to verify whether the expected SSEs can be observed after generating the 3D model. The map shows how distant bases in 3D structure of the ssDNA are from each other. Orange areas in the highlighted SSEs in the distance map show the formed base pairs of the 3D structure and have a distance between 1.54 Å and 1.92 Å. The bases that formed the asymmetric interior loop have a distance between 13.10 Å and 15.00 Å (dark blue color in Figure 2C). The generated 3D structure with the formed SSEs was used as basis for the MD simulation.

### 2.2. MD Analysis

To examine the behavior of the modeled E2-specific aptamer structure and the binding behavior of E2 within the binding pocket, an MD simulation with GROMACS was performed. In this study, two MD simulations were carried out and compared with each other. First, an aptamer simulation was carried out without ligand, called aptamer-free (AptF) structure simulation. Afterwards, the same aptamer was simulated with ligand, called E2-aptamer complex (E2AptC) simulation. Both simulations had the same starting point and were compared to identify structural differences. To study the stability of the E2-specific aptamer structures over the 25 ns simulation time, various analytical methods were employed.

**Figure 3:**
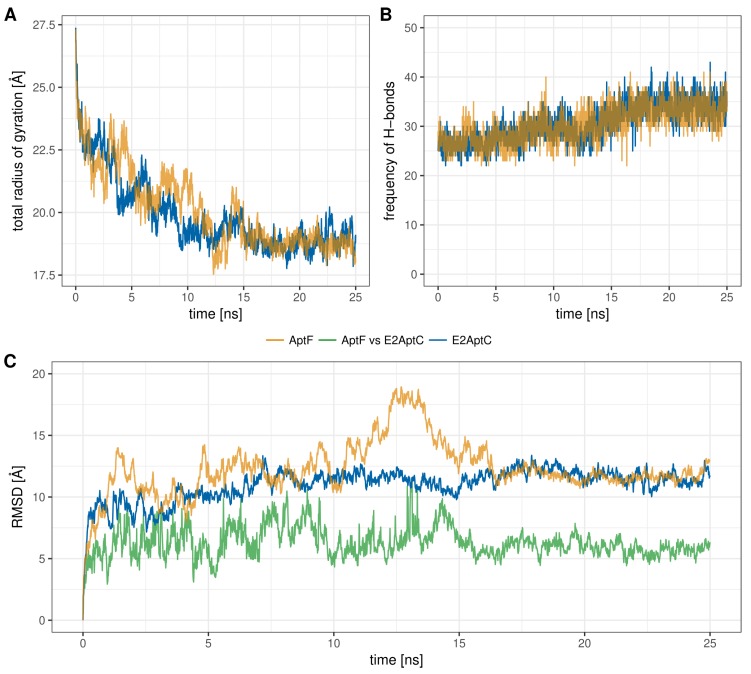
Different structure stability analyses versus time plots during the 25 ns MD simulation for AptF (orange line) and E2AptC (blue line): (**A**) radius of gyration; (**B**) number of H-bonds within the aptamer structure; and (**C**) structural deviation (RMSD calculation) over the course of MD simulation with respect to the starting structures of AptF and E2AptC. The green line shows the structural deviation between AptF and E2AptC from the respective time step. AptF, aptamer-free; E2AptC, E2-aptamer complex; RMSD, root mean square deviation.

The radius of gyration ($R_{g}$) is an established measurement to identify structural stability and compactness of modeled structures [40]. The $R_{g}$ plot in Figure 3A shows the comparison of the structures AptF and E2AptC. Both structures showed stabilization after a simulation time of 15 ns. From this point on, both structures are considered at equilibrium. The average $R_{g}$ value of AptF structure is 20.10 ± 1.56 Å. In the case of AptF, these value remained virtually unchanged with an average $R_{g}$ value of 19.88 ± 1.48 Å.

Similar to the $R_{g}$ value, the number of intramolecular formed H-bonds can also contribute to a basic understanding of the aptamer structure stability [41]. The total number of H-bonds from AptF and E2AptC are shown in Figure 3B. The number of H-bonds increased over the course of both simulations. The average number of H-bonds was 30.3 ± 3.5 for AptF and 30.6 ± 3.7 for AptF. The minimal number of H-bonds was 22 for both simulations and the maximal number was 41 for AptF and 43 for E2AptC. Both structures have a similarly high compactness, based on the small $R_{g}$ value and an approximately constant number of bonds.

Dynamic stability of E2-specific aptamers over the simulation of 25 ns was analyzed using root mean square deviation (RMSD). The plot describing the RMSD of the AptF and E2AptC states during the simulation time of 25 ns is shown in Figure 3C. Additionally, the structural deviation between AptF and E2AptC is also displayed in Figure 3C. The maximal RMSD of AptF was observed to be 18.93 Å. The average RMSD of AptF was 12.28 ± 2.17 Å. From approximately 11 to 15 ns, the AptF structure began to bend strongly. This brought the 5${}^{\prime }$-end and the hairpin very close together. The RMSD values were between 11.89 Å and 18.93 Å during this time; after this period, the two aptamer structure elements unbent again. The $R_{g}$ was also subject to a higher fluctuation between 17.54 Å and 21.04 Å during this period. In the case of E2AptC, the RMSD was observed by a maximum of 13.38 Å with an average RMSD value of 10.99 ± 1.27 Å. The equilibrium in the AptF structure is achieved after a simulation time of 16.25 ns (Figure 3C). In contrast, the state of stability in E2AptC structure already began after 15 ns. To examine the similarity between the AptF and E2AptC structures, each state of the respective simulation was compared and analyzed using RMSD. The average RMSD was 6.31 ± 1.21 Å and has a maximum RMSD of 11.51 Å (Figure 3C, green line).

**Figure 4:**
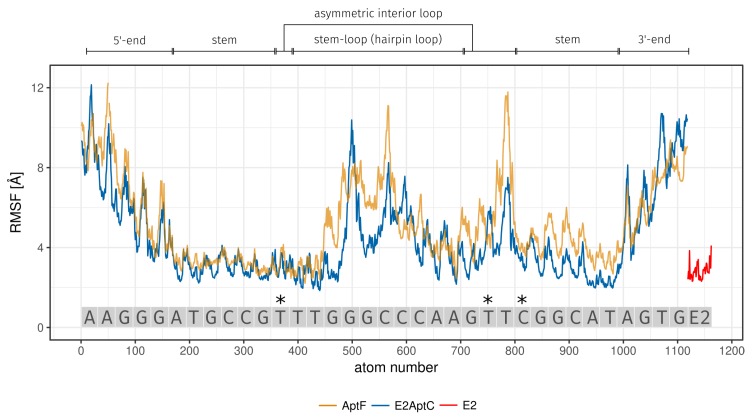
RMSF of aptamer and E2 atoms during 25 ns MD simulation. RMSF comparison between AptF (orange line) and E2AptC (blue line). E2 is a part of E2AptC MD simulation and is depicted as a red line. The various secondary structures of the E2-specific aptamer are shown above the plot and the binding bases of E2 are highlighted with the asterisk symbol (${}^{*}$). AptF, aptamer-free; E2, 17β-estradiol; E2AptC, E2-aptamer complex; RMSF, root mean square fluctuation.

The calculation of the root mean square fluctuation (RMSF) can be used to evaluate the flexibility differences between atoms. The RMSF characterizes structural flexibility, was calculated for the 25 ns trajectory of E2-specific AptF and E2AptC, and is displayed in Figure 4 and Table 1. The bases of the E2 binding site are a part of the asymmetric interior loop (T12 and T24) and the stem region (C26). The RMSF values for the single bases are 3.18 Å (T12), 5.20 Å (T24), and 4.20 Å (C26) for AptF trajectory. In the case of E2AptC, these values decrease to 3.10 Å (T12), 4.31 Å (T24), and 3.61 Å (C26) (Table 1). E2 is a part of the E2AptC MD simulation and exhibits a small movement within the E2-specific aptamer, represented by the small RMSF value of 2.81 Å (Figure 4, E2 is depicted as red line).

### 2.3. E2-Interaction Analysis

**Figure 5:**
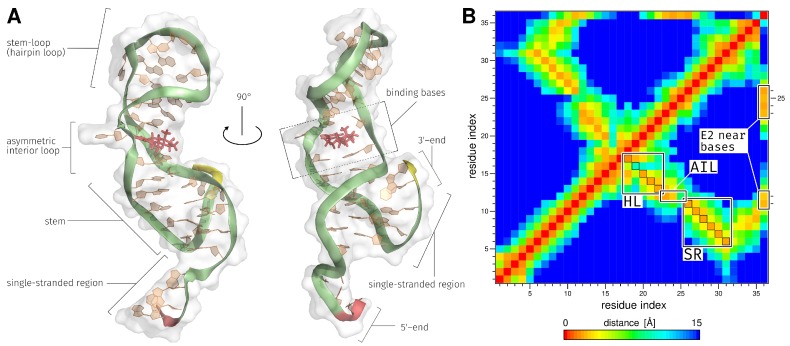
(**A**) Final model of the E2AptC obtained from 25 ns MD simulation, with formed SSE. The 5${}^{\prime }$-end is shown in red within the structure and the 3${}^{\prime }$-end in yellow. E2 is shown as stick model and in red color. For improved visibility, all water molecules and ions have been removed. (**B**) Distance map of the smallest distance between every pair of bases (residue index 1–35) and E2 (residue index 36) of the last time step of MD simulation. Bases that are very close to each other are displayed in red. The SSE and the E2 near bases are framed in black. Within the framed areas, the orange and cyan fields show the formed SSEs with a distance range between 1.54 Å and 10.00 Å. The bases in the AIL region have a distance between 1.92 Å and 8.46 Å. The orange fields of the E2 near bases have a distance between 1.92 Å and 2.69 Å. AIL, asymmetric interior loop; E2, 17β-estradiol; E2AptC, E2-aptamer complex; HL, hairpin loop; SR, stem region; SSEs, secondary structure elements.

MD simulations allow observing the binding behavior between aptamer and ligand over a defined period of time. A binding analysis shows at each individual time step the bases of the aptamer involved in the binding and the chemical binding types that contributed to the binding of E2. The structural analysis has shown that the SSE were maintained and reinforced during 25 ns MD simulation (Figure 5A,B). In particular, the bases in the asymmetric interior loop have contracted and exhibit now a maximal spatial distance of 8.46 Å (Figure 5B). The ligand binding takes place inside the asymmetric interior loop of the E2-specific aptamer (Figure 5A and the corresponding animation is provided in Video S2). From the distance map it became clear that the bases G11, T12, G23, T24, T25, and C26 have the smallest distance to E2 (Figure 5B). G11 and C26 are a part of the stem region and have a distance of 2.31 Å and 1.92 Å to E2. All four bases of the asymmetric interior loop have a small distance to E2. These include T12 with a distance of 2.69 Å, G23 with 2.31 Å, T24 with 1.92 Å, and T25 with 2.31 Å. 

**Figure 6:**
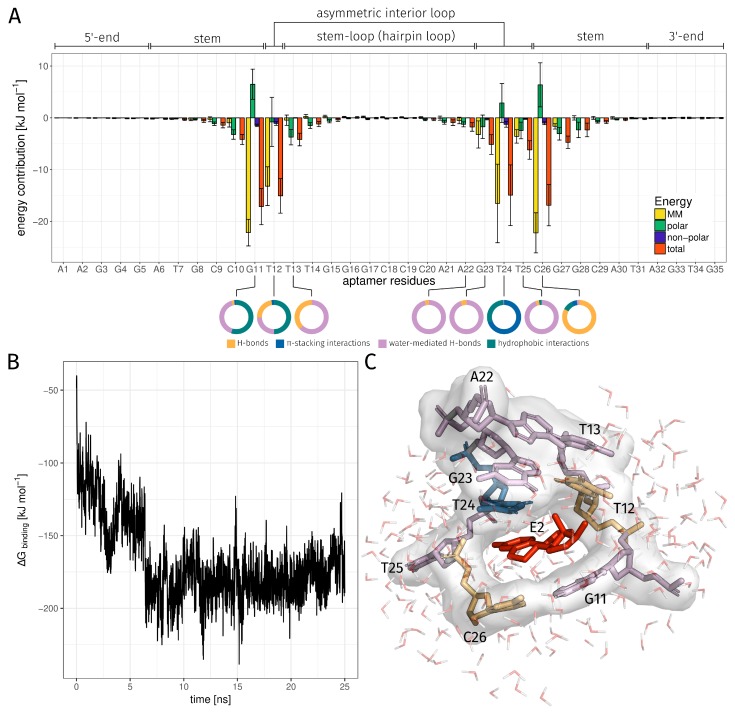
(**A**) Contribution of the aptamer bases to free energy. The various secondary structures of the E2-specific aptamer are shown above the plot. Small donut charts below the energy contribution plot show the distribution of different interactions of the bases interacting with E2. (**B**) Binding energy ΔG analyses versus time for E2AptC. (**C**) Structural overview of all detected E2 binding bases. The color of binding bases corresponds to the color of the main specific interaction that was formed with E2. The bases T12 and T25 mainly formed H-bonds, the base T24 π-stacking interactions, and the other bases water-mediated H-bonds to E2. E2AptC, E2-aptamer complex; E2, 17β-estradiol is depicted as stick model in red; H-bonds, hydrogen bonds.

To understand aptamer-ligand association at the molecular level, it is helpful to determine the aptamer bases that interact with the ligand. The bases strongly interacting with E2 are G11, T12, T24, and C26. The total free binding energy between E2 and these four bases is in the range from −17.15 $\mathrm{kJ}\cdot {\mathrm{mol}}^{‒1}$ to −14.95 $\mathrm{kJ}\cdot {\mathrm{mol}}^{‒1}$ and contributes the majority of binding energy (Figure 6A). From an energetic point of view, these bases are the most important interaction partners of E2 and in Figure 6C it can be seen that these bases directly surround E2. The base T25 contribute a total energy value of −6.20 ± 1.79 $\mathrm{kJ}\cdot {\mathrm{mol}}^{‒1}$ and exhibits a higher energy than the other four. This base is also very close to E2 and binds E2 non-specifically with phosphate backbone of the aptamer (Figure 6C). T25 had a small part in the binding of E2 and was involved in 10.84% of time in binding (Table 2). Interaction analysis using the PLIP tool has shown that these four bases also play an important role in the formation of non-covalent interactions (Table 2 and Figure S1). The distribution of the interactions at the respective positions can be seen below the energy contribution plot (Figure 6A). This shows that the binding of E2 by base G11 mainly consist of hydrophobic interactions and water-mediated H-bonds. Base T12 mainly binds with hydrophobic interactions, water-mediated H-bonds, and H-bonds. Base T24 binds with π-stacking interactions and base C26 with H-bonds. In addition to these four dominant bases, other bases were detected that also had non-covalent interactions with E2. These include T13, A22, G23, and T25 and their energy values are summarized in Table S1. The average binding energy $\Delta G_{\mathrm{binding}}$ also obtained from the MM/PBSA calculation of the E2AptC was −168.32 ± 0.57 $\mathrm{kJ}\cdot {\mathrm{mol}}^{‒1}$ and the corresponding trajectory can be seen in Figure 6B. Furthermore, the corresponding components of the average binding energy $\Delta G_{\mathrm{binding}}$, the van der Waals energy $\Delta E_{\mathrm{vdw}}$ were calculated with −145.17 ± 0.39 $\mathrm{kJ}\cdot {\mathrm{mol}}^{‒1}$, electrostatic energy $\Delta E_{\mathrm{ele}}$ with −24.31 ± 0.22 $\mathrm{kJ}\cdot {\mathrm{mol}}^{‒1}$, polar solvation energy $\Delta G_{\mathrm{polar}}$, and non-polar solvation energy $\Delta G_{\mathrm{non}-\mathrm{polar}}$ with −12.85 ± 0.02 $\mathrm{kJ}\cdot {\mathrm{mol}}^{‒1}$. In the time range from 1 ns to approximately 6 ns, the binding energy $\Delta G_{\mathrm{binding}}$ decreases strongly from −40.09 $\mathrm{kJ}\cdot {\mathrm{mol}}^{‒1}$ to −210.10 $\mathrm{kJ}\cdot {\mathrm{mol}}^{‒1}$ (Figure 6B). A minimum binding energy $\Delta G_{\mathrm{binding}}$ between the aptamer and E2 of −238.45 $\mathrm{kJ}\cdot {\mathrm{mol}}^{‒1}$ was achieved at 15 ns. Figure 6C shows E2 and a small section of the aptamer structure. A total of eight bases have been identified that have formed non-covalent interactions with E2. The colors of the bases correspond to the most frequently detected specific integration types per base.

**Figure 7:**
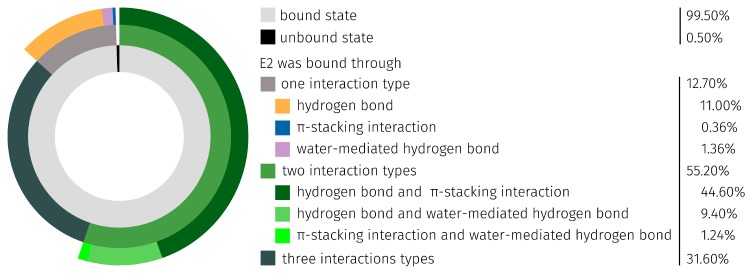
Donut chart of detected specific interactions between E2 and the E2-specific aptamer during the 25 ns MD simulation.

To determine which types of interaction occur most frequently and in which combination, these are divided according to their occurrence (Figure 7), but non-specific interactions were not included in the donut chart. In over 99% of the simulation time, E2 was bound by the aptamer. The ligand was bound in more than 12% by only one, in approximately 55% by two, and in over 31% by all three types of detected interactions. E2 is bound in 11% of the simulation time by a single H-bond. The combination of the interaction types H-bond and π-stacking was dominant in the binding of E2 by the aptamer (over 44%). Least present were the water-meditated H-bond (1.36%), π-stacking interaction (0.36%), and the combination of these (1.24%).

The main aptamer binding bases T12 and C26 formed 98.21% of all detected H-bonds and T24 formed 89.27% of all detected π-stacking interactions with E2 (Table 2 and Figure 7). Furthermore, it was possible to observe how E2 was bound through water-mediated H-bonds. In total, 1479 of these bounds were generated through the surrounding water molecules and all eight binding bases. In 0.50% of the total 25 ns MD simulation, E2 was not bound through the observed three different binding types formed by the E2-specific aptamer.

**Figure 8:**
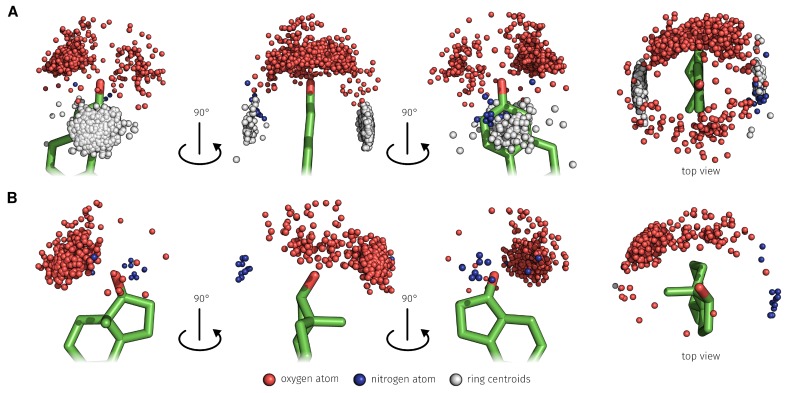
Distribution and different perspectives of the detected base interaction atoms during the 25 ns MD simulation that formed O–H⋯O (red atoms) and N–H⋯O (blue atoms) H-bonds and ring centroids representing the π-stacking interaction (gray pseudo atoms). (**A**) The focus is on the 3-position of E2. (**B**) The focus is on the 17-β-position of E2. E2, 17β-estradiol.

To determine which bases played a major role during the binding process it was checked how frequently each base at positions 3 and 17-β has formed the respective interactions (Table 3). At the 3-position of E2, H-bonds are dominant over the water-mediated H-bonds and at the 17-β-position, the water-mediated H-bonds are dominant. C26 formed 97.64% of 2415 H-bonds from type O–H⋯O to the 3-position of E2 (Figure 8A). Furthermore, 89.27% of 1976 of the π-stacking interactions from base T24 to the A-ring of E2 were formed (Figure 8A). T12 formed 95.21% of 438 H-bonds from type O–H⋯O to the 17-β-position of E2 (Figure 8B). The corresponding geometric characteristics such as bond lengths and angle between donor and acceptor atoms and ring centers of the π-stacking interactions are described in the Supplementary Materials (Figure S2).

**Figure 9:**
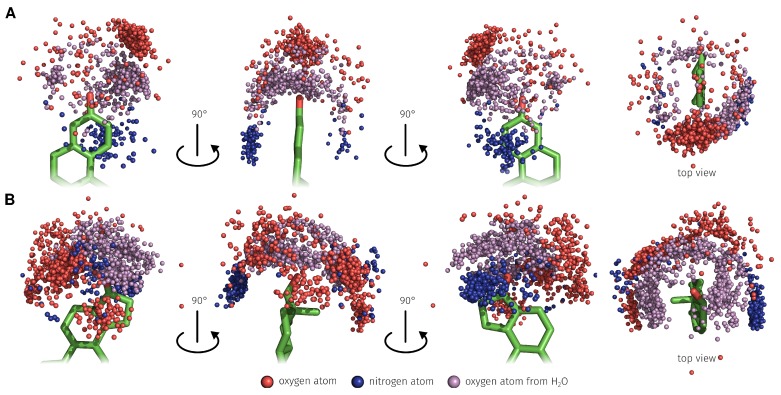
Distribution and different perspectives of the detected base interaction atoms during the 25 ns MD simulation that formed O–H⋯O–H⋯O (red, purple, and red atoms) and N–H⋯O–H⋯O (blue, purple and red atoms) water-mediated H-bonds. (**A**) The focus is on the 3-position of E2. (**B**) The focus is on the 17-β-position of E2. E2, 17β-estradiol.

Through use of explicit solvent in the MD simulation, the formation of water-mediated H-bonds between E2 and the aptamer was possible. In the 25 ns MD simulation, a total of 1479 water-mediated H-bonds between aptamer and E2 were detected. 494 (33.40%) was formed on the 3- and 985 (66.60%) on the 17-β-position of E2 (Figure 9). On the 3-position, 77.13% of the water-mediated H-bonds interactions were of type O–H⋯O–H⋯O and 22.87% of type N–H⋯O–H⋯O. On the 17-β-position, 67.31% of the water-mediated H-bonds interactions were of type O–H⋯O–H⋯O and 32.69% of type N–H⋯O–H⋯O.

## 3. Discussion

The aim of this study was to detect the interactions between the E2-specific aptamer and E2 and to describe the binding characteristics. For this purpose, a 25 ns MD simulation was performed and the trajectory was used for the analysis. It was found that, in addition to the H-bonds and π-interactions, the water-mediated H-bonds are also important for the binding of E2. The results shown in this article are based on theoretical methods and thus should be handled with care as no experimental evaluation of the binding site of E2 has been carried out.

### 3.1. Generation of the ssDNA Aptamer Structure

The DNA aptamer structure was created using the coarse-grained modeling method [33]. With the MMB (also known as RNABuilder), it was possible to predict and model the base pairs of the DNA structure based on secondary structure, since this method can also be used for RNA modeling. The MMB was mainly used for the modeling of RNA structures [33,42], but was also successfully used for the modeling of ssDNA structures [43,44]. The predicted SSEs could be completely modeled with the MMB (Figure 2B,C). This structure was used as starting point for the presented MD simulation study. The MMB coarse-grained modeling method is an excellent option to model the unknown E2-specific 3D DNA aptamer structure [16] from the 2D structure and was also used in other studies [45,46]. Hilder and Hodgkiss [35] described in their study the DNA structure prediction from the E2-specific DNA aptamer which was done with the RNAcomposer [47]. The RNAcomposer is based on fragment libraries generated from known structures for the assembly of RNA molecules [47]. The RNAcomposer was not used in this study to predict the DNA-aptamer 3D structure, as it is only recommended for predicting RNA structures [47]. Therefore, we decided to use MMB because this method is also suitable for the generation of DNA 3D structures [33]. In addition to the MMB and RNAcomposer, there are other methods that are capable of predicting single-stranded nucleotide tertiary structures. Further methods are necessary, because the crystallographic structure determination of aptamers and ligand-aptamer complexes still poses a challenge [48,49,50].

### 3.2. MD Analysis

To determine the possible structural differences between the AptF and E2AptC, two independent all-atom MD simulations were carried out. The simulations were carried out with GROMACS [51] using the force field AMBER 99 [52]. GROMACS is, in addition to other simulation software packages such as CHARMM [53], NAMD [54], and AMBER [55], established software for sophisticated simulation systems. AMBER 99 [52] and AMBER 99 parmbsc0 [56] force fields have proven to be particularly suitable for nucleotide simulation as they contain an improved ion parameter for the interaction with nucleotides. In addition to this force field for nucleotides, there are also force fields AMBER 03 [57], GROMOS 53A6 [58], and CHARMM27 [59], which can be used for the simulation of nucleotides. Simulations of ssDNA were also carried out with the force fields listed above [60,61,62]. A successful ligand-aptamer complex simulation was carried out by Lin et al. in 2012 to examine the induced-fit mechanism [63] that was initiated by the binding of L-argininamide through DNA aptamer [64]. The structure comparison between AptF and E2AptC revealed a slight conformational change. This change indicates an induced-fit mechanism that was caused by E2 binding. To validate these findings, it would be necessary to carry out further MD simulations, which includes the following steps: (I) short aptamer simulation without ligand; (II) positioning of the ligand near the potential bond bases and simulation of the complex formation; and (III) simulation of the ligand-aptamer complex. The binding properties and dynamics of the E2 ligand within protein [65,66] and DNA [35,67] structures have been analyzed mainly in 2017 by MD simulation studies.

### 3.3. E2-Interaction Analysis

In addition to the structural analysis and the observation of the ligand E2 inside the aptamer structure, an intensive examination of the formed interactions between E2 and ssDNA was carried out. PLIP was used for the interaction analysis, as it is suitable for the detection of interactions between protein and ligand as well as for the nucleotide ligand complex. With PLIP it was possible to detect H-bonds, π-stacking, water-mediated H-bonds, and hydrophobic interactions between the aptamer and E2 for each time step of the MD simulation. In addition to PLIP, the interactivity analyses between the aptamer and E2 would have been possible with Arpeggio as an alternative [68].

Interaction analyses of E2–target MD simulations have already been performed, but these studies have only considered a selected time step [35,66,67]. Water-mediated H-bonds were only analyzed in the study of Jereva et al. [66] and not in the interaction analyses between E2 and DNA [35,67]. In general, the interaction analysis of a trajectory is useful because it allows the separation of selective and non-selective ligand binding partners and to observe a possible change in these. In this study, the main interacting bases T12, T24, and C26 of the E2-specific aptamer could be determined and differentiated from the non-selective bases. However, the non-selective bases also play an important role and support E2 to remain the position and direction inside of the aptamer.

**Figure 10:**
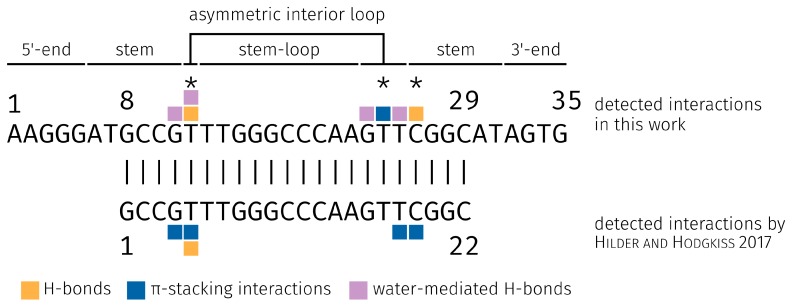
Comparison and local sequence alignment of the E2-specific aptamers. Both aptamers were identified by Alsager et al. [16]. The detected interaction bases of the aptamers and their different interaction types with E2 from the study of Hilder and Hodgkiss [35] and from this work are highlighted. The various secondary structures of the E2-specific aptamer are shown above the plot and the main binding bases of E2 are highlighted with the asterisk symbol (${}^{*}$). H-bonds, hydrogen bonds.

Hilder and Hodgkiss [35] identified the four bases G4, T5, T18, and C19 as the predominant binding partners of E2 in their study (Figure 10). The aptamer of Hilder and Hodgkiss [35] is a subsequent of the aptamer presented here, but originates from the same underlying study [16]. The sequence core area that can bind E2 is the same, only the 5${}^{\prime }$-end and 3${}^{\prime }$-end are different. Therefore, the base G1 of the short 22-base aptamer sequence [16,35] is the base G8 of the longer 35-base aptamer sequence and so on. In our study, we have chosen the 35-base aptamer sequence, because the 5${}^{\prime }$- and 3${}^{\prime }$-end can potentially be used for immobilization on different substrates [16]. It is advantageous to examine the dynamics of the 5${}^{\prime }$-end and 3${}^{\prime }$-end to ensure that it will not interact with E2 or otherwise be integrated into the bond and will not cause any changes in the conformation of the asymmetric interior loop.

Different energy based approaches can be used to examine the binding strength between target and ligand. In the study of Hilder and Hodgkiss [35], the free energy perturbation (FEP) method [69] was used to calculate the binding strength. We used the molecular mechanics Poisson–Boltzmann surface area (MM/PBSA) method [70,71] to estimate the bond strength between the aptamer and E2. The method FEP is accurate but computationally expensive. Method MM/PBSA, on the other hand, is also predestined for calculating the free binding energy of systems involving in water and is faster than the FEP method [72]. Molecular mechanics energy $E_{\mathrm{MM}}$ is the sum of bonded $E_{\mathrm{bonded}}$ (composed of bond, angle, dihedral and improper interactions), electrostatic $E_{\mathrm{ele}}$ and van der Waals energy $E_{\mathrm{vdw}}$ [70] and has the most impact on the energy contribution between E2 and the aptamer. The polar solvation energy $G_{\mathrm{polar}}$ and non-polar solvation energy $G_{\mathrm{non}-\mathrm{polar}}$ only contribute slightly to free binding energy. For the four main E2 binding bases G11, T12, T25, and C26 the calculated total energy values based on the MM/PBSA method are 45.40 ± 7.10% lower than the energy values reported by Hilder and Hodgkiss [35]. In general, the same bases interact with E2 (Figure 10) and the energy values of both methods show a significantly increased binding strength of the four bases to E2, in contrast to the remaining bases.

We discovered that the bases G11, T12, T24, and C26 formed a π-stacking interaction to the A-ring of E2 during the simulation (Figure S1). However, base T24 plays a special role in the binding of E2, as it was most dominant in the binding. Hilder and Hodgkiss [35] were able to observe the formation of π-stacking at bases G4, T5, T18, and C19. The base T18 of the 22-base aptamer sequence [35] corresponds to the base T25 in the 35-base aptamer sequence, with this base no π-stacking interaction could be detected. It has been demonstrated that the bases G and T can form π-stacking interactions to the A-ring of E2 [67].

The comparison shows that the interactions with E2 were formed by the two aptamers in the same region (Figure 10). It was found that the two bases T12 and C26 form the H-bonds and play an important role in binding E2 through the 35-base aptamer. However, in the study of Hilder and Hodgkiss [35], it was found that only base T5 formed a H-bond. The base T5 of the 22-base aptamer corresponds to the base T12 of the 35-base aptamer. In general, H-bonds at the positions 3 and 17-β of the E2 are possible [66].

Differences in the aptamer structures, as well as the E2 position within the aptamer, between the study of Hilder and Hodgkiss [35] and our study, may have been caused by the use of different force fields. Hilder and Hodgkiss [35] used the force fields CHARMM36 and CHARMM27 for the MD simulation and in our study we used the AMBER 99 force field. Comparative studies have shown that the use of the force fields CHARMM27 and AMBER 99 in MD simulations has led to a different behavior of DNA structures [73,74]. Point mutations at the detected binding bases could be used to determine the importance of a single base for the binding of E2. Furthermore, it would be possible to study the changes of non-covalent interactions between E2 and the aptamer and to compare them with previous results. With the introduced point mutation it is possible to examine the structural change of the aptamer and to determine possible effects for the binding of the ligand. It is possible that the ligand changes its position inside the pocket or that only weak interactions are formed with the ligand. Additionally, other interactions between E2 and the aptamers can be detected, as long as a uniform detection method is used, to make the results comparable.

It has been established that water-mediated H-bonds play important roles in target–ligand binding complexes [75,76]. For the first time, water-mediated H-bonds were examined and evaluated in a ssDNA–ligand interaction MD simulation. Figure S1 shows that all bases around E2 formed water-mediated H-bonds with them. The bases G11, T12, and T25 form the most frequent water-mediated H-bonds to E2. G11 as a part of stem region of the E2-specific aptamer G11 is part of the E2-specific aptamer stem region and has a minor role in relation to H-bonds and π-stacking interactions. This study shows that base G11 makes an important contribution to the binding of E2 at the 3-position. The following base T12, on the other hand, already has an important role in the binding of E2, as it also forms the H-bonds to the 3-position of E2. The additional, formed water-mediated H-bonds supports the strong anchoring of E2 within the asymmetric interior loop. On the side of the 17-β-position of E2, the bond is enhanced by the water-mediated H-bonds, originating from the base T25. This base is also part of the asymmetric interior loop, but in terms of H-bonds and π-stacking interactions only plays a minor role. Through the π-stacking interactions at the A-ring, E2 is more limited in its movements at the 3-position than at the 17-β-position. This allowed more selective H-bonds to form at the 3-position of E2. However, on the 17-β-position, E2 is more variable, so several intermediate states, the water-mediated H-bonds, are required to stabilize the ligand. However, the binding of E2 with the water-mediated H-bonds originating from T25, is strongly supported by the asymmetric interior loop. The water molecules play an important role in the binding of E2 through the aptamer, as the possible water-mediated H-bonds strongly contribute to the binding of E2 within the aptamer. It has been established that hydrophobic interactions also play an important role in target–ligand binding complexes [77]. E2 is a molecule with low polarity because of its dominant hydrocarbon skeleton. Therefore, the hydrophobic interactions were considered in the interaction analysis (Figure S1). Four bases G11, T12, T24, and C26 are involved in the formation of hydrophobic interactions. The main bases therefore took a double and triple role in binding of E2. The base T12 takes part the H-bonds of direct interactions, water-mediated H-bonds and hydrophobic interaction. The base T24 was able to form interactions via π-stacking and hydrophobic interactions to E2. Furthermore, the base C26 was able to form the interaction types H-bonds and hydrophobic interactions to E2. It is conceivable that the bases in the studies of Hilder and Hodgkiss [35,67] may also play a manifold role in the binding of E2, as is the case with amino acids in the study of Jereva et al. [66].

A experimental in vitro validation to identify the binding site by mutation studies is planned and would be a crucial step to experimentally validate the results of this study. Further, a possible step to evaluate the developed workflow is to use an existing ligand-aptamer complex from the PDB and compare it and the binding site with the structure produced by our method. This procedure could also be carried out with the workflow of Hilder and Hodgkiss [35] to compare the results of both methodologies. When executing the workflow it should be known that the used aptamer is able to bind a specific molecule. It is not recommended to model a aptamer with known sequence and perform the binding studies with a arbitrary ligand. The same applies to a binding study where the ligand is in focus but no specific aptamer is available. Reliable results can only be obtained with sufficient knowledge about both the aptamer and the ligand.

## 4. Conclusions

The results suggest that the interaction analysis, in combination with a target–ligand MD simulation, paves the way for the understanding of the intrinsic dynamics of a ligand binding through a target structure. For these studies, we have developed a general workflow that allows analyzing the interactions between a ligand and an aptamer structure starting from a known aptamer sequence. In this study, the binding behavior of E2 by means of the E2-specific aptamer was successfully analyzed and an interplay of interactions was observed. It was found that dominant bases (T12, T24, and C26) exist in the asymmetric interior loop of the aptamer that formed various non-covalent bonds with E2. Alsager et al. and Svobodová et al. did not analyse the binding interaction between E2 and the aptamer. Therefore, it was not possible to include these studies in the comparison of the binding interaction. It was possible to confirm the binding bases found by Hilder and Hodgkiss with our study [35]. Besides the dominant bases, there are other bases with a small distance to E2 (G11, T12, G23, and T25). These are involved in the formation of interactions and thus contribute to the binding of the ligand within the aptamer structure. Mainly H-bonds, π-stacking interactions, and water-mediated H-bonds were analyzed, but also the non-selective hydrophobic interactions were considered. The use of an explicit solvent in the MD simulation, enables the determination of water-mediated H-bonds. These not only occur in protein E2 [66], but also in aptamer E2 interactions and thus contribute to ligand bindings. Furthermore, the importance of H-bonds and π-stacking interactions for the binding of E2 through proteins or DNA aptamers was demonstrated. Because of the predominant hydrophobic and rigid backbone of E2, hydrophobic interactions are key for the assessment of the E2 binding. Nonetheless, the focus of this study was on specific interactions (H-bonds and π-stacking interaction) and water-mediated H-bonds, as these are the strongest non-covalent bond types [38]. The identification of the binding bases and the comparison with the study by Hilder and Hodgkiss [35] allows a targeted design of the aptamer structure to achieve an optimization of the binding specificity. Furthermore, we could confirm that the two aptamer ends 5${}^{\prime }$ and 3${}^{\prime }$ are not interacting with E2 and are therefore free to be used for immobilization on potential substrates.

## 5. Methods

**Figure 11:**
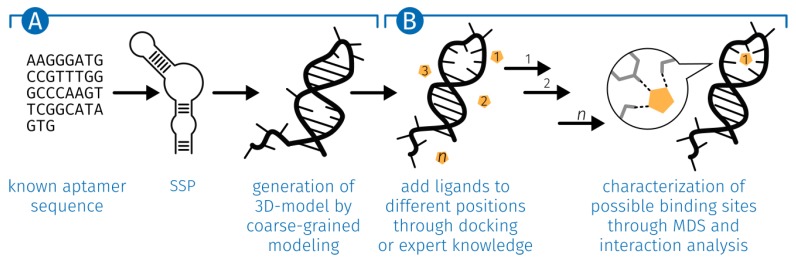
Schematic overview of the developed workflow for detailed analysis of ligand-aptamer interactions. (**A**) Starting from a known ligand-specific aptamer sequence and the prediction of the secondary structure, the 3D structure is modeled. (**B**) The modeled 3D structure is the basis for the MD simulation. First, the ligand is integrated within the structure through docking or expert knowledge. After the MD simulation, the resulting structures of the individual time steps are examined for possible binding sites and the interactions formed are detected. MDS, molecular dynamics simulation; SSP, secondary structure prediction.

For the study of the interactions between E2 and the specific aptamer a structure is essential. Since there is no 3D structure, a workflow was developed that generates a 3D structure from the DNA sequences (Figure 11).

### 5.1. Generation of the ssDNA Aptamer Structure

The E2-specific DNA aptamer (5${}^{\prime }$-AAGGGATGCCGTTTGGGCCCAAGTTCGGCATAGTG-3${}^{\prime }$) structure was modeled with the MMB [33] command line tool v2.17 (Flores et al., Uppsala, Sweden) and the last state of the generated structures was used as starting point structure for the MD simulation. The structure was defined as DNA type and was instantiated as sequence in single letter code taken from Alsager et al. [16]. To define the base pairings, a base interaction scale factor of 800 was selected, which enforces specific base pairings. Furthermore, the default temperature value was set to 280 K for the MD parameters in the MMB tool. For the E2-specific aptamer structure, two areas with base pairs were defined, which were derived from the SSP (identified by Alsager et al. [16]). The first base pair area was defined from residue identifier 6 to 11 and from 31 to 26 as the stem region and the second base pair area was defined from residue identifier 13 to 15 and from 22 to 20 as the stem region from hairpin. The default MD parameters provided by MMB [33] were chosen.

### 5.2. MD Simulation

MD simulations track the changes of a macromolecular system and are useful to examine the E2-ssDNA interaction [35,67]. The simulation systems were prepared by embedding the last MMB model in cubic water boxes with approximately 125,700 water molecules, 34 Na${}^{+}$ atoms and a simulation box size of 156 Å${}^{3}$ for AptF and E2AptC. The *editconf* module of GROMACS was used for creating boundary conditions and *genbox* for solvation. The spc216 water template was chosen in this configuration. Each explicit MD simulation system contained a total number of approximately 126,900 atoms. A minimum distance of 30 Å was kept between the surface of the E2-specific DNA aptamer and the borders of the simulation box. The simulations were carried out using GROMACS [51] v5.1.3 (Abraham et al., Stockholm, Sweden) with the all-atom AMBER 99 (Wang et al., California, San Francisco) [52] force field and TIP3P model for water. In addition, the ligand E2 has been integrated into the simulation study of E2AptC and the E2 parameter files obtained with AmberTools16 [78] were converted to GROMACS [51] format using ACEPYPE [79]. Afterwards, the energy of the simulation system was minimized to eliminate structural deformations with 1000 steps of steepest descent algorithm or until the maximal force threshold (<1000 $\mathrm{kJ}\cdot {\mathrm{mol}}^{‒1}\cdot {\mathrm{nm}}^{‒1}$) was achieved. For both simulation systems, AptF and E2AptC, the temperatures were subsequently increased from 0 to 298.15 K and the pressure from 0 to 1 bar during the equilibration period under periodic boundary conditions. After the equilibrium was achieved through NVT and NPT a production phase of 25 ns was carried out for both AptF and E2AptC simulation systems. All MD simulations were performed with Intel(R) Xeon(R) CPU E5-2690 v2 @ 3.00GHz (Intel Corporation, Santa Clara, CA, USA) and the GPU NVIDIA Tesla K40c (Nvidia Corporation, Santa Clara, CA, USA) with Ubuntu 14.04.5 LTS. The trajectories of the MD simulations were examined with GROMACS [51] tools (e.g., RMSD, RMSF, H-bond frequency and Radius of gyration) and were visualized using R v3.4.3 [80] (R Core Team, Wien, Austria). All the pictorial structure presentations were prepared using PyMOL Molecular Graphics System v1.8.6.0 (Schrödinger, LLC, New York City, NY, USA).

The free binding energies of the complex between the aptamer and E2 were analyzed during the 25 ns MD simulations with g_mmpbsa tool for GROMACS [81]. This approach based on the molecular mechanics Poisson–Boltzmann surface area (MM/PBSA) and is a common method for calculating the binding free energy between target and ligand. The calculation of the free energy always follows the same formula [81] and the corresponding equations have already been presented in numerous publications [82,83,84]. The temperature was adjusted to 298.15 K according to the MD simulation parameters. All other parameters of g_mmpbsa have been kept at the default settings.

### 5.3. E2-Interaction Analysis

Detection of non-covalent interactions between the E2-specific aptamer and E2 ligand was performed with the PLIP [38] command line tool v1.3.4 (Salentin et al., Dresden, Saxony, Germany) on all extracted trajectory models of the whole MD simulation. The additional command line argument for interactions of molecules with DNA (dnareceptor) was used for the detection, which is available in the development branch. The resulting information was used for the extraction of the binding bases and for the investigation at the atomic level. Furthermore, it was possible to study the interaction for each time step of the MD simulation.

## Figures and Tables

**Table 1:** Comparison of the RMSF values between AptF and E2AptC. The SSEs of the aptamers, their corresponding bases with identifier, E2, and their RMSF values in Å are displayed. AptF, aptamer-free; E2, 17β-estradiol; E2AptC, E2-aptamer complex; RMSF, root mean square fluctuation; SSEs, secondary structure elements.

Region	Base Range	RMSF [Å] of AptF	RMSF [Å] of E2AptC
5${}^{\prime }$-end	A1 to G5	7.34	6.43
stem	A6 to G11 and C26 to T31	3.72	3.02
asymmetric interior loop	T12 and G23 to T25	5.61	3.99
hairpin loop	T13 to A22	5.14	4.24
3${}^{\prime }$-end	A32 to G35	6.57	6.96
3*E2 binding site	T12 (asymmetric interior loop)	3.18	3.10
	T24 (asymmetric interior loop)	5.20	4.31
	C26 (stem)	4.20	3.61
E2	-	-	2.81

**Table 2:** Overview of the detected interactions between E2 and the bases of E2-specific aptamer during the 25 ns MD simulation. The relative (rel.) frequency of the detected interactions H-bonds, π-stacking interactions, water-mediated H-bonds, and hydrophobic interactions are displayed. The last column shows the relative frequency of the respective base, which forms an interaction with E2 during the whole 25 ns simulation time. H-bonds, hydrogen bonds.

Binding Bases	H-Bond rel. [%]	π-Stacking int. rel. [%]	Water-Mediated H-Bond rel. [%]	Hydrophobic int. rel. [%]	Total rel. [%]
DG11	1.00	0.80	15.44	21.08	33.44
DT12	17.68	1.92	19.04	38.64	60.96
DT13	0.32	0.00	0.48	0.00	0.68
DA22	0.04	0.00	0.96	0.00	1.00
DG23	0.24	0.00	3.92	0.00	4.16
DT24	0.04	70.56	0.56	29.76	76.44
DT25	0.36	0.00	10.24	0.28	10.84
DC26	94.36	5.76	2.68	11.16	98.00

**Table 3:** Overview of the detected interactions between 3- and 17-β-position during the 25 ns MD simulation. The absolute (abs.) and relative (rel.) frequency of the detected H-bonds and water-mediated H-bonds are displayed. The last column lists the total number of interactions per E2 position. E2, 17β-estradiol; H-bonds, hydrogen bonds.

E2 Position	H-Bond abs./rel. [%]	Water-Mediated H-Bond abs./rel. [%]	Total abs./rel. [%]
3	2415/84.65	494/33.40	2909/67.15
17-β	438/15.35	985/66.60	1423/32.85
∑	2853/100.00	1479/100.00	4332/100.00

## Supplementary Materials

The following are available online, Video S1: Animation of formed structure by MMB and the corresponding distance matrix, Figure S1: Different interaction analyses versus time plots during the 25 ns E2AptC MD simulation, Figure S2: General geometric characteristics of H-bonds, π-stacking interactions, water-mediated H-bonds, and hydrophobic interaction, Table S1: Binding energy components and binding free energy for binding bases of the aptamer, Video S1: 25 ns MD simulation trajectory videos of the E2-aptamer complex, Data S1–4: Data from PLIP of the detected four interaction types as CSV file format, Data S5: Trajectory from the 25ns E2-aptamer complex MD simulaiton as PDB file format. The PDB file contains: E2, E2-specific aptamer, and all solvent molecules and ions 10 Å around E2.

## Abbreviations

The following abbreviations are used in this manuscript:

ACPYPE	AnteChamber PYthon Parser interfacE
AIL	asymmetric interior loop
AptF	aptamer-free
E2	17β-Estradiol
E2AptC	E2-aptamer complex
FEP	free energy perturbation
GROMACS	Groningen Machine for Chemical Simulations
H-bond	hydrogen bond
HL	hairpin loop
MMB	MacroMoleculeBuilder
MD	molecular dynamics
MM/PBSA	molecular mechanics Poisson–Boltzmann surface area
NPT	constant number (N), pressure (P), and temperature (T)
NVT	constant number (N), volume (V), and temperature (T)
PLIP	Protein–Ligand Interaction Profiler
R${}_{g}$	Radius of gyration
RMSD	root mean square deviation
RMSF	root mean square fluctuation
SELEX	systematic evolution of ligands by exponential enrichment process
SR	stem region
ssDNA	single stranded DNA
SSE	secondary structure element
SSP	secondary structure prediction
